# ChainRank, a chain prioritisation method for contextualisation of biological networks

**DOI:** 10.1186/s12859-015-0864-x

**Published:** 2016-01-05

**Authors:** Ákos Tényi, Pedro de Atauri, David Gomez-Cabrero, Isaac Cano, Kim Clarke, Francesco Falciani, Marta Cascante, Josep Roca, Dieter Maier

**Affiliations:** Hospital Clínic-Institut d’Investigacions Biomediques August Pi i Sunyer (IDIBAPS), Research Institute, Universitat de Barcelona, C/Villarroel 170, 08036 Barcelona, Spain; Departament de Bioquimica i Biologia Molecular, Facultat de Biologia-IBUB, Universitat de Barcelona, 08028 Barcelona, Spain; Unit of computational Medicine, Center for Molecular Medicine, Department of Medicine, Karolinska Institute and Karolinska University Hospital, SE-171 76 Stockholm, Sweden; Centro de Investigación en Red de Enfermedades Respiratorias (CibeRes), 07110 Palma de Mallorca, Spain; Integrative Systems Biology, University of Liverpool, L69 3BX Liverpool, UK; Biomax Informatics AG, D-82152 Planegg, Germany

**Keywords:** Biological networks, Protein-protein interaction, Data integration, Filtering, Computational biology, Bioinformatics, Systems biology, COPD

## Abstract

**Background:**

Advances in high throughput technologies and growth of biomedical knowledge have contributed to an exponential increase in associative data. These data can be represented in the form of complex networks of biological associations, which are suitable for systems analyses. However, these networks usually lack both, context specificity in time and space as well as the distinctive borders, which are usually assigned in the classical pathway view of molecular events (e.g. signal transduction). This complexity and high interconnectedness call for automated techniques that can identify smaller targeted subnetworks specific to a given research context (e.g. a disease scenario).

**Results:**

Our method, named ChainRank, finds relevant subnetworks by identifying and scoring chains of interactions that link specific network components. Scores can be generated from integrating multiple general and context specific measures (e.g. experimental molecular data from expression to proteomics and metabolomics, literature evidence, network topology). The performance of the novel ChainRank method was evaluated on recreating selected signalling pathways from a human protein interaction network. Specifically, we recreated skeletal muscle specific signaling networks in healthy and chronic obstructive pulmonary disease (COPD) contexts. The analysis showed that ChainRank can identify main mediators of context specific molecular signalling. An improvement of up to factor 2.5 was shown in the precision of finding proteins of the recreated pathways compared to random simulation.

**Conclusions:**

ChainRank provides a framework, which can integrate several user-defined scores and evaluate their combined effect on ranking interaction chains linking input data sets. It can be used to contextualise networks, identify signaling and regulatory path amongst targeted genes or to analyse synthetic lethality in the context of anticancer therapy. ChainRank is implemented in R programming language and freely available at https://github.com/atenyi/ChainRank.

**Electronic supplementary material:**

The online version of this article (doi:10.1186/s12859-015-0864-x) contains supplementary material, which is available to authorized users.

## Background

Canonical pathways are widely used tools to represent signal transduction and molecular networks. They generally rely on literature-based information, mostly derived from hypothesis-driven experiments collected in exceedingly diverse contexts, encompassing a large variety of experimental conditions (e.g. different species, cell-types/tissues, diseases) and/or in-vitro models. Multiple layers of information (e.g. direction of a signalling event, type of interactions or cartoon graphics) make literature-based pathways a highly accepted and convenient source of information in biological research. However, the emergence of high-throughput technologies has shown several limitations of the approach.

By incorporating non-hypothesis based interactions, high-throughput methods have revealed many previously unrecognised pathway components [1–3]. Moreover, different studies have shown high interconnectedness of signalling pathways indicating larger complexity than the conventional separate representation of molecular events [4, 5]. Furthermore an increasing amount of evidence suggests the dependence of biological, cellular and disease outcomes on the complex of interactions between genes, proteins and other molecules [6] which is rarely addressed in pathway databases. Consequently, it is currently apparent that the classical pathway approach is too simplistic to properly describe complex cellular events [7–9].

With advances in high-throughput technologies an increasing number of genome scale association data became available. This scenario facilitates the construction of data-driven biological networks, integrating experimental data, e.g. on protein-protein interactions (PPI), gene regulation and metabolic interactions, offering a systems approach to model molecular events [10]. However, these networks are too large for human interpretation and their context specific origin is often unaccounted in databases. Therefore, filtering these networks and identifying subnetworks that are important in a certain context (e.g. disease/health, tissue/cell) are major challenges that make up an active field of research.

An appealing approach for relevant subnetwork identification is to model the flow of biological information (e.g. cell signalling) using chains of interactions. In the case of protein signalling this means that every protein in a chain can modify the consequent protein, transmitting a biological signal (the alternative term “path” is avoided here to prevent confusion with signalling pathways). Multiple alternative chains which allow to traverse from a start to an endpoint may exist within a network. Following this logic Scott et al. [11] successfully developed an algorithm to identify protein signalling cascades in a protein network for pathway discovery purposes. They used interaction reliability and functional enrichment based scoring to calculate the significance of the chains. They showed that this technique has a potential in recovering known pathways in yeast, however, their algorithm lack context specificity and is not publicly available. Other methodologies use gene expression data to get more context specific results. Teku et al. [12] developed a filtering method to identify a core T cell network using the immunome interactome. They used a co-expression based weighting of the interaction network to compute the significance of the links. However, expression based specificity is not the only factor defining the importance of a protein in an added context. Functional module identification methods based on topological structures of unweighted PPI networks are another active area of research. For example lately, Liekens et al. [13] introduced a solely network based methodology for gene prioritisation using an integrated interaction network. According to the assessment of the authors, this method, despite its exclusively topology based search algorithm, was reported to outperform earlier gene prioritisation algorithms based on data fusion of heterogeneous data sources [13]. Recent reviews on pathway discovery approaches provide further examples for the interested readers [14, 15].

Here, we present ChainRank, an enhanced search and prioritisation tool that allows combining multiple biological evidences (e.g. topology, experimental molecular data from expression to proteomics and metabolomics, literature evidence, meta-analysis results, phenotype association) as scores. Similarly to the work of Scott et al. [11], our method uses a chain based network search algorithm to retrieve chains linking user defined start and end nodes, e.g. biomarkers associated with a disease state. In this work, we show that combining different context specific and topological scores together with a chain based search approach that simulates real interaction mechanisms – instead of focusing on individual biological elements or their associations – can improve the prediction of underlying pathway mechanisms. We introduce a framework over the search algorithm that can incorporate multiple user defined scores and thus is able to contextualize search results to e.g. disease states or tissues. Furthermore, we show that this framework can evaluate the combined effect of these scores to simulate complex phenotypes, e.g. tissue specific effects of a certain disease. According to our knowledge this is the first method relying on a chain based approach that is able to incorporate various scores and combine them and this is the first study showing the effect of combining different scores.

To assess ChainRank, we evaluated three scores (topological, tissue specific and disease state specific) to prioritise chains within a PPI network and evaluate them against known gold standard signalling pathways. We focused our analysis on muscle dysfunctions in chronic obstructive pulmonary disease (COPD) because of its specificity to a distinctive tissue, and also because of its clinical relevance. We introduce two complex, biologically motivated scores that we created integrating multiple differential expression studies as well as expression, protein and metabolite data to describe tissue- and disease wise importance of the network proteins. We also present a score describing topological importance and show the combined effects of the developed scores. Evaluating the precision and recall of finding gold standard (GS) proteins in our top scoring results, we show a considerable increase in precision with comparably good recall rate, compared to a simulated random scoring. Furthermore we show that combining different scores can further improve the performance of the prioritisation. The results demonstrate that our method can effectively identify pathway elements in a context specific manner. Potential use cases are the identification of disease specific networks, assessment of pathway interactions, simulation of the spread of perturbing effects amongst networks (mode-of-actions) and the elucidation of mechanistic relations between biomarkers.

Our method is implemented in the popular R framework and freely available at https://github.com/atenyi/ChainRank.

## Methods

**Fig. 1:**
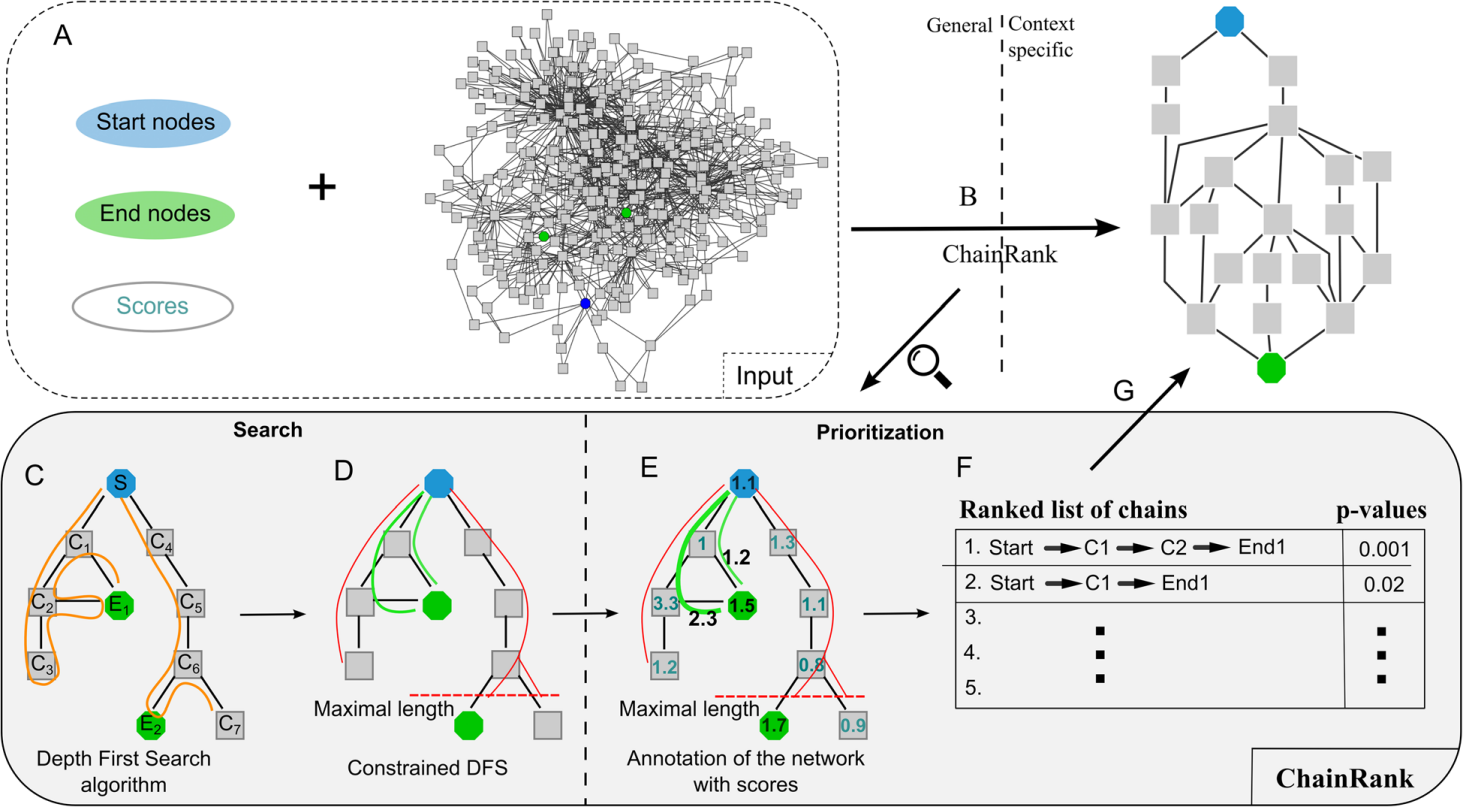
Schematic overview of the ChainRank method (**a-b**) and its workflow (**c-f**). **a** The input parameters of the algorithm are the investigated research targets (start and end nodes), a network and the defined scores. **b** The ChainRank method produces a context specific subnetwork specific to the research targets. **c** The method is based on a depth-first search (DFS) algorithm that traverses the network from the starting node and explores a branch as far as possible before backtracking. **d** DFS is constrained to search only chains that has a maximal length or smaller. **e** Network nodes are annotated with scores. Chain scores then calculated by the sum of the scores of chain elements, normalized by the length of the chain. **f** The significance of the chains are calculated using the chain scores. **g** Most significant chains are selected and used for the construction of a context and target specific subnetwork

The ChainRank method consists of two main steps. The first step searches for all chains connecting start and end nodes in a network (Fig. 1c-d). For example given a start node S which interacts with node C1 which interacts with proteins C2 and E1 (Fig. 1c), as such we define two chains between S and E, namely S-C1-E1 and S-C1-C2-E (Fig. 1f). The next step involves annotating the network nodes with scores and computing the chain scores and p-values to provide a ranking and selection (Fig. 1e-f).

### Chain search

The chain search step is used directly to evaluate all potential chains connecting start and end nodes within the initial network. This task translates to the “all simple paths” graph theoretical problem [16] that seeks to find all simple (non-cyclical) paths between two vertices. A graph of n vertices contains n! simple paths which makes a brute force search an NP hard problem. However, for signalling and gene regulatory networks the biological relevance of connections between two entities diminishes with increasing relative distance, i.e. the given distance relative to the shortest distance [17, 18]. Therefore, the problem can be addressed by introducing a depth limit for the search that is greater or equal to the distance of the shortest path linking the start and end nodes. This problem can be optimally solved by a depth limited depth first search (DFS) algorithm. The basic DFS algorithm traverses the network from the starting node and explores a branch of the network before backtracking (Fig. 1c). Using a depth limit the search is halted if a chain would exceed a specified *k* maximal length (depth limit) which is defined as the number of nodes a chain contains (Fig. 1b). This algorithm has Ο (b^k^) time complexity, where *b* is the branching factor of the graph and due to its exhaustive nature it finds an optimal solution within the depth limit *k* [19]. We implemented a recursive version of this algorithm and extended it to be able to search simple paths amongst multiple start and end nodes. Chains connecting start and end nodes are stored and serve as the output of the algorithm. The method was implemented in R programming language. The pseudo code of the algorithm is detailed in the Additional file 1: Text S1.

### Scoring and prioritisation using p-values

In order to create a general prioritisation framework, we introduced the concept of element scores. Such scores are mapped to network nodes and describe a specific property of a biological entity that the node represents. This score can include both topological and biological characteristics (e.g. the connectivity of a node or tissue specific expression of the protein/gene that the node represents or experimental support for a protein-protein interaction) (Fig. 1) and a node can hold one or more separate scores. We used these measures to characterize the interaction chains. Our aim was to maximize the overall score of the nodes in a chain, therefore we used the sum of their element scores to calculate the chain scores. Furthermore, to exclude length based biases we normalized this score by the length of the chain to get the final chain score, thus *S* = ∑_*i*_^*l*^*s*_*i*_/*l* where *S* denotes the chain score, *l* is the length of the chain and *s*_*i*_ is the score of the i^th^ element of the chain.

Certain research situations involve several biological contexts, e.g. disease effects on specific tissue. To address such needs, we introduced the concept of combined scores. We introduced three different strategies to combine the scores: (i) Combined scores are calculated as the weighted product of the normalized element scores mapped to a node, using the formula *c*_*k*_ = ∑_*j*_^*n*^*w*_*j*_*s*_*kj*_, where *c*_*k*_ is the combined score of the *k*^th^ node, *n* is the number of scores, *s*_*j*_ is the *j*^th^ element score normalized to the range [0,1] and *w*_*j*_ is the weight corresponding to the *j*^th^ score, (ii) the filtering strategy pre-filters the chains using a threshold for the score *s*_1_, and then it re-ranks the filtered chains with score *s*_2_ and (iii) the intersection strategy keeps only those chains that are under a specified threshold for all the selected scores.

To evaluate the chain scores, we calculate the significance of the chains. We simulated random networks, constructed by shuffling the weights and edges of the initial network, while preserving the vertex degrees. For a given chain with score *s*, its score p-value is defined as the percentage of top-scoring chains in random networks that have score *s* or higher [11].

We also use the score p-value to generate the list of prioritised chains. Depending on the application a score p-value cut-off can be utilized to select the most significant chains or alternatively the top scoring *n* chains can be selected. Assembling the filtered chains allows for the reconstruction of a subnetwork that is specific to the start and end nodes and to the context the score defines.

### Evaluation and performance

To evaluate a computational method one can either apply a measure of stability by cross-validating multiple runs or, ideally, derive precision and sensitivity information from comparison against a standard of truth. As described in the introduction there is a lack of context aware pathways which could be used as standard of truth. In order to evaluate the results of the ChainRank we therefore validate our method on two levels. First, the significance of the chain scores is evaluated. Second, a reference pathway is used as a validation set and the enrichment of its members in the top results or the ranked chains is assessed for the evaluation. This validation set is referred to as the gold standard (GS). To judge the stability of the method we compute the precision and recall of the top *n* chains or alternatively use a p-value cut-off. For the validation, positives (P) are defined as the validation elements represented in the input network but not included in the start and end proteins. To determine the precision, the occurrence of the validation set elements are counted in the top chains (excluding start and end proteins), i.e. the true positives (TP), while non-validation set elements represents the false positives (FP). Thus, *Precision* = *TP*/(*TP* + *FP*) and *Recall* = *TP*/*P*. Due to the lack of well-defined GS, reaching high precision values is a highly challenging task. Therefore to represent our results in a more informative way we defined the metric of improvement. To compute the improvement of a ranking we simulate a random score, i.e. we perform a random sampling from the chains to select the top results. Then, we compute *ovement* = (*Precision of ranking*)/(*Precision of random ranking*).

## Results

**Fig. 2:**
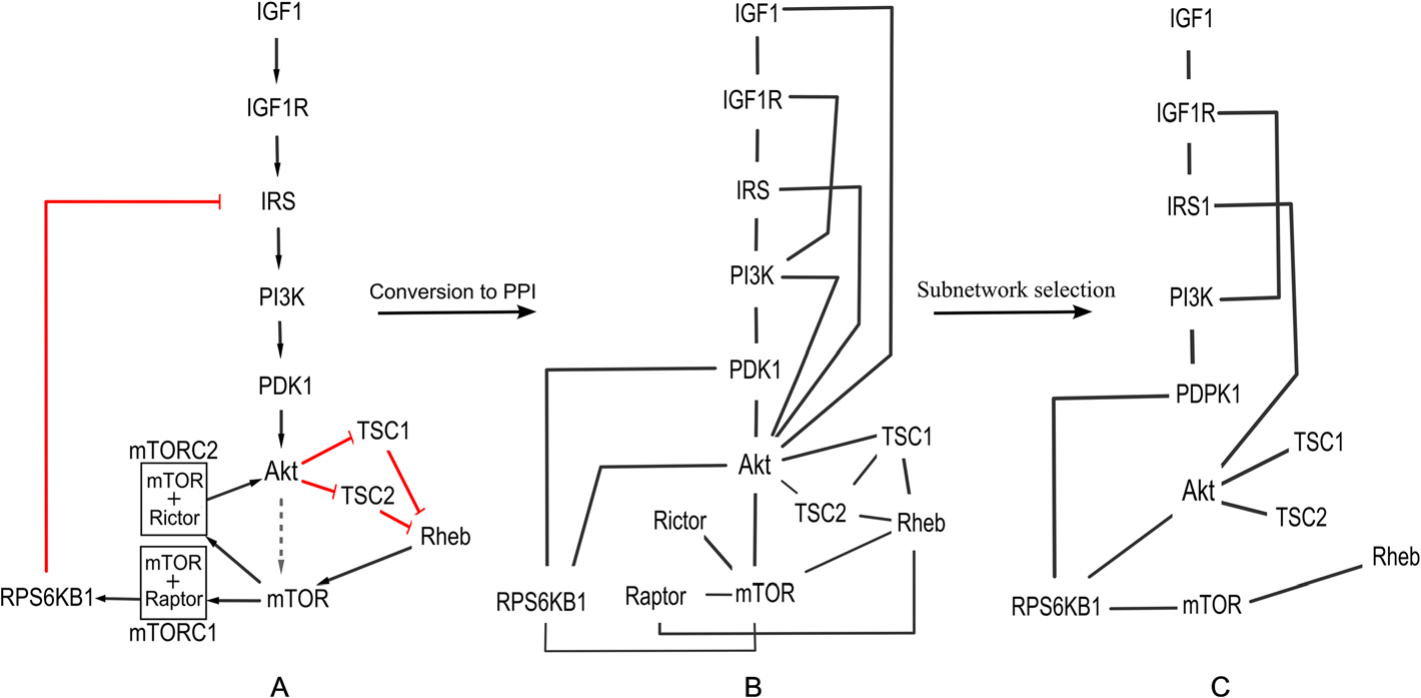
Changes in the representation of IGF-Akt pathway. **a** shows the protein synthesis regulation related part of IGF-Akt pathway [21]. In this pathway representation relations of proteins are well annotated with their directionality, type of interaction, etc. **b** shows that during manual conversion of the canonical pathways to PPI network representation (by retrieving all PPI interactions between the fixed set of e nodes) this kind of information is lost and only undirected edges represent physical binding between proteins. Moreover, the structure of the pathway is altered by extra edges and some connections are missing. **c** shows the effect of the network selection which removes redundant edges but retains most of the nodes

In order to assess the performance of our method we studied its applicability in protein interaction network based pathway reconstruction. We specified the domain of interest to muscle dysfunctions in chronic obstructive pulmonary disease (COPD) because of its specificity to a distinctive tissue, its clinical relevance as well as the wealth of literature mining and experimental data available for our analysis [20]. We designed two application cases, each with a specific GS pathway (Table 1.). First, we aimed to recreate a subnetwork of the IGF-Akt pathway [21] describing regulation of protein synthesis, an important aspect of muscle remodelling (Fig. 2a). In the second case, our goal was to represent the disease specific involvement of parts of a canonical signalling pathway. We used disease specific varieties of the canonical MAPK pathway: the EGF-PI3K and ROS-TGFa-EGFR pathways (Additional file 1: Table S1, Fig. S7), that are based on literature mining for COPD related signal transduction events [20]. We note that evidence for the involvement of these specific parts of the GS pathways is not excluding potential involvement of additional parts. For the evaluation we selected specific chains from these pathways defined by start and end proteins that we refer as gold standards (Table 1).

**Table 1:** Overview of the networks used in the evaluation process and the gold standards. Gold standard representation is shown in the original PPI network and in the selected networks. *Edges* signify the number of edges connecting GS *Nodes* in the network

Application case	Network properties		Gold standard (GS)	Start protein	End protein	GS representation	
	Edges	Nodes				Nodes	Edges
Human PPI network	61872	10167	IGF-Akt pathway	-	-	13	20
			COPD specific MAPK	-	-	21	34
Muscle specific case	847	308	IGF-Akt pathway	IGF1	RPS6KB1	9	10
COPD related case	544	152	COPD specific MAPK	EGFR	SRF, CREBBP, ELK1, MYC	11	8

### PPI network

For the investigations we utilized the complete human PPI network as the input network. At the time of the analysis it contained 1.6 million protein interactions that were collected and merged from different publicly available databases and integrated into the COPD knowledge base [20]. We quality filtered this network by including only those interactions that were supported by at least one piece of experimental evidence (in contrast to purely computationally predicted ones). This resulted in a PPI network of around 10,000 nodes and 62,000 interactions (Table 1).

### Subnetwork selection and performance

Within this general PPI network we are only interested in the specific subnetwork that potentially connects our start and target set, here determined by the endpoints of our selected gold standard pathways. In order to retrieve this subnetwork as starting point for the ChainRank method, we applied the BioXM knowledge management environment network search tool [22]. This tool is based on a heuristic breadth-first search algorithm, allows nodes to be preferred or penalised based on their connectivity and it retrieves those nodes in the input network that have the potential to link targeted nodes within a *k* maximal distance. Consequently, with this step we omit those unnecessary nodes and edges that does not lead to any targeted endpoints in a *k* maximal length chain. Therefore, we decided to set the *k* distance cut-off for the breadth first search centered on the distance between the start and the target in our reference GS path. Furthermore, Baudot et al. [18] showed that canonical signal transduction pathways are enriched for highly connected protein hubs; therefore, we set the algorithm to encourage the integration of canonical interactors preferring highly connected proteins. We generated two subnetworks (IGF-Akt proximity and MAPK proximity subnetworks, Table 1.). Because heuristic subnetwork generation methods introduce an element of variability, we evaluated its effect by creating further networks with different parameterisation and analysed them in terms of their overall influence on the ChainRank results which was not significant (Additional file 1: Table S2 and S4).

As an alternative to the heuristic network selection step the ChainRank method could be used to evaluate all potential chains of a given maximal length within the overall network. However, the corresponding computational requirements quickly become prohibitive as longer chains are explored in dense networks (see Chain search). Runtime of the chain search for the muscle-specific network (314 nodes, 865 edges) with a maximal length of 8 is 14.5 min on a 2.4 GHz processor, finding more than 9000 chains. In addition, we note that the size of the network that the ChainRank method can process in realistic time depends strongly on the network complexity (more runtime data on different networks is available in Additional file 1: Table S3).

### Evaluation of the input network

In order to set a realistic gold standard (GS) for the evaluation we analysed the changes in the canonical GS during its manual conversion to a PPI representation and then the effect of the network selection (Fig. 2). In canonical pathways relations of proteins are manually selected and well annotated with their directionality, type of interaction, etc. During the conversion of these pathways to a PPI network representation the annotation is lost and only physical interaction without pre-selection are depicted. Therefore edges appear/disappear during the conversion and protein complexes become individual, interacting nodes. These findings show the high complexity of searching in PPI networks and demonstrate that the exact recreation of a canonical pathway cannot be the ultimate metric of the evaluation process but rather the relative improvement between unranked and ranked searches.

### Scores

As mentioned in the introduction there are several methods that use gene expression data to investigate domain specific traits. While ChainRank is able to incorporate gene expression scores, here we focus on more complex scores to represent localisation or disease relevance. We also introduced a topology based score.

1. Localisation score: To show the capabilities of the method in tissue-specific filtering we created a muscle specificity score. Using this prioritisation with the ChainRank method would result in those interaction chains that contain mostly muscle specific proteins being highly ranked. To create this score we collected publicly available gene expression measurements from Gene Expression Omnibus (GEO) [23], studying a large amount of different conditions in different tissues. We compared the mean variability of the genes’ expression value in muscle to their mean variability in the rest of the body. Genes with highly variable expression levels under different conditions in muscle but lower variability in other parts of the body receive higher scores while genes that are not typically variable in muscle or are variable throughout all tissues receive lower score. The corresponding proteins were mapped to genes to be applicable for PPI network based analyses. Details on the included data sets and the exact methodology can be found in the Additional file 1: Text S2.
2. Relevance score: This score describes the relevance of a protein in a specific biological process — in this case a disease. To generate a disease specific score we used studies that investigated the effect of COPD on skeletal muscle and other mechanisms that related to this disease. The selected studies incorporated diverse experimental paradigms such as proteomics, metabolomics and gene expression. From these studies we extracted all genes or proteins (depending on the type of analysis) that were shown to be significantly changed in the disease context. Then we computed the score by counting how many times a gene/protein occurs with high significance in any of these study results. The first study we utilized investigated the training effect on the muscle of COPD patients [24] integrating measurements of gene expression, metabolism and protein carbonylation [25–27]. In addition, as part of this research study, the effect of angiogenesis on gene expression in young (<30 year) and elderly (>60) persons was examined (detailed in the Additional file 1: Text S3). Finally, an analysis on inactivity-induced wasting in mouse glycolytic muscle was used to construct the score [28] (detailed in Additional file 1: Text S3). We used HomoloGene [29] to find homologous human genes for the mouse genes and we mapped the genes to the related proteins in all the studies.
3. Connectivity score: We used a topology based score to characterize the degree centrality of the proteins in the network. We reversed the degree centrality to compute the score, thus *Connectivity score*(*v*) = |*dc*(*v*) − max(*dc*(*V*))| + 1, where *dc*(*v*) is the degree centrality of *v* ∈ *V* vertice. This score is a good measure to distinguish between general hub like proteins with high degree centrality (and thus with low scores) and specific proteins with lower degree centrality (and thus high scores).

To test the sensitivity of our algorithm to different scores that explain similar biological phenomena, we introduced two additional scores from external data sources. As an alternative to Localisation score, we retrieved the Tissue Specificity (TS) score from the Human Protein Atlas [30], which corresponds to the score calculated as the fold change to the second highest tissue (for further information see Additional file 1: Text S4). As an alternative to Relevance score we created the Fold change (Fc) score, which we retrieved from a recent publication that reported RNA-seq data for 98 COPD subjects and 91 controls [31]. Score was computed as Fc = log2(COPD/control), where COPD and control is the gene expression value of the signed group.

### Evaluation of the scores: distribution, correlations and the length of the chains

**Fig. 3:**
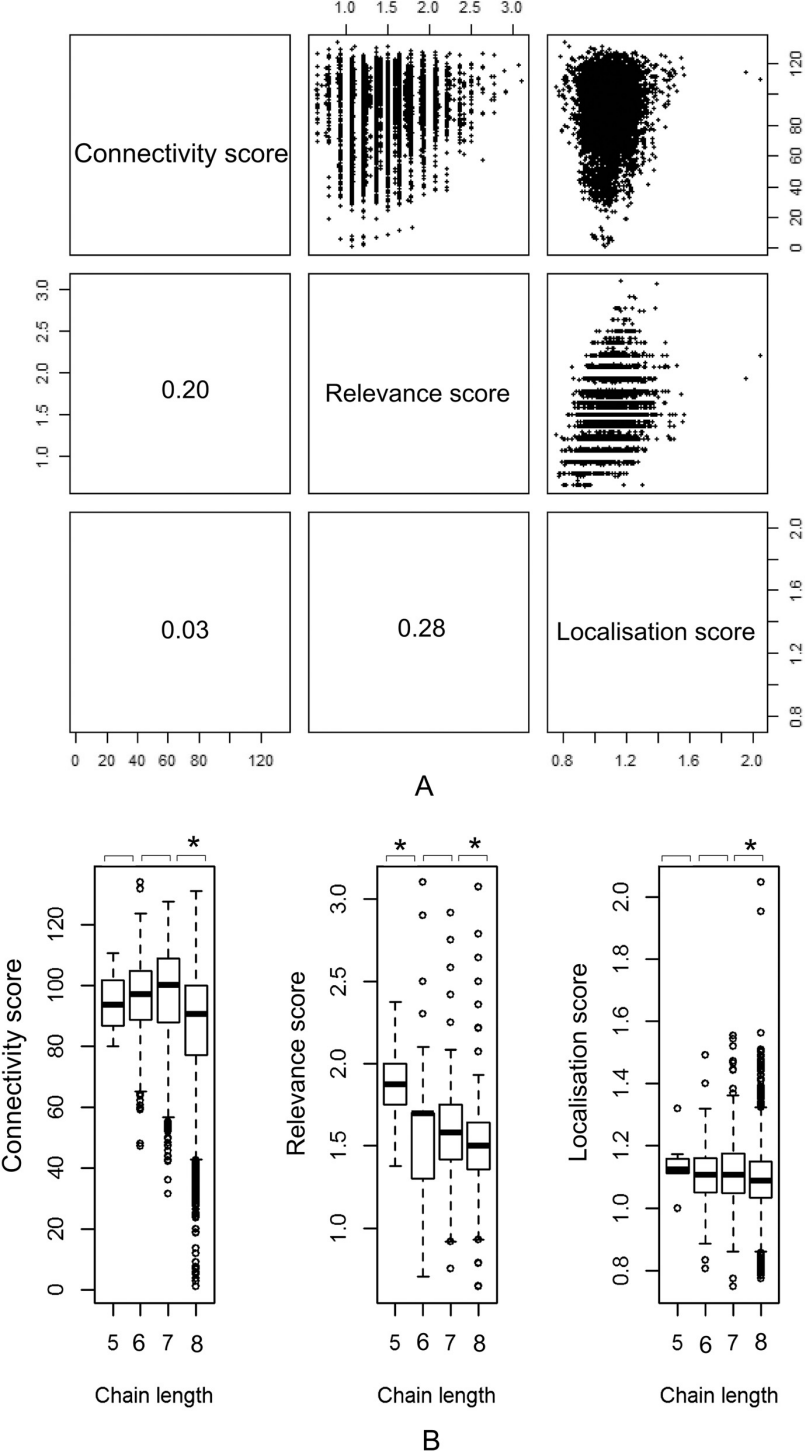
Statistical evaluation of the scores. **a** shows the correlation between the chain ranks, correlation values are indicated in the lower triangle. **b** shows the relation of the length of the chains to the chain scores. Statistical significance between the different length chains’ scores is indicated (*p ≤0.05)

In order to check for the independence of our selected scores we examined their correlation and their relation to the length of the chains. We used the IGF-Akt proximity subnetwork, with maximal length 8 for this analysis. Figure 3a shows that the expression and relevance scores show a slight correlation which can be explained by the fact that in this case the relevance score (among other aspects, such as protein carbonylation and metabolites) includes data on gene expression in muscle tissue. Therefore, although the relevance rank is based on experiments with specific environmental factors, the expression data is expected to show some correlation with the general muscle expression measurements. The other variables are uncorrelated, therefore we can assume that the different ranks explain different properties of the chains. We found that normalisation of the chain scores by the number of chain nodes removes most of the length dependency (Fig. 3b). We note that different topological properties of the networks might have effect on the connectivity scores’ length dependence. Furthermore, we showed that the distribution of scores in the generated subnetworks (Subnetwork selection and performance, Table 1.) represents well the distribution of scores over the whole PPI network (Additional file 1: Figure S1).

### Evaluation of the performance of the ChainRank method

**Fig. 4:**
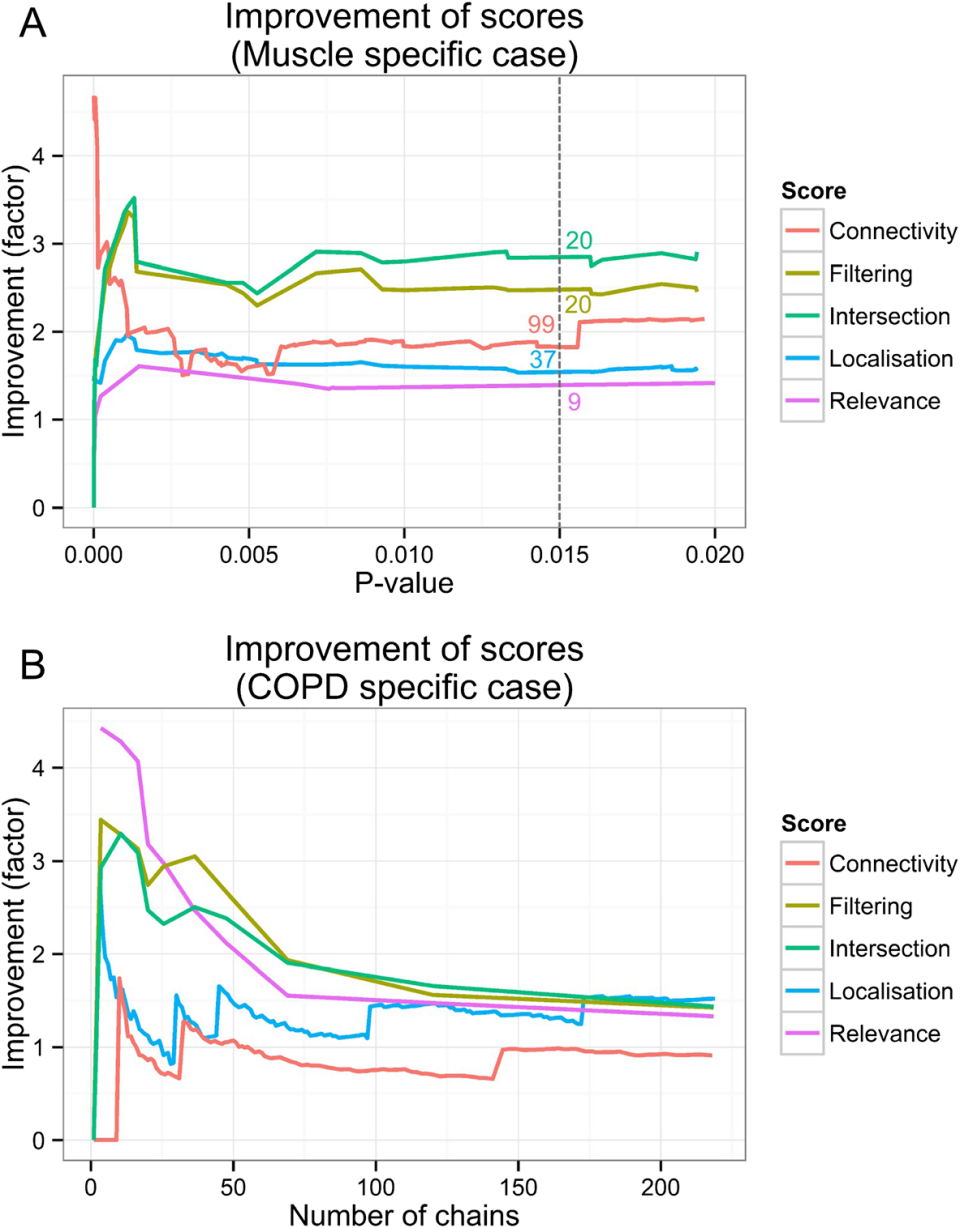
Improvement of the different scores. **a** Muscle specific case: Intersection is defined as the common chains that have both a p-values ≤0.05 with Connectivity and Localisation score. These chains are shown with their Localisation score p-values. For Filtering chains with Connectivity score p-values ≤0.05 were selected, re-ranked and evaluated by Localisation score. Number of chains are indicated at p =0.015 for each score. **b** COPD specific case, Intersection here is defined as the common chains in the top quartile of chains ranked by Localisation score and Relevance score. These chains are shown with the number of top chains ranked by Relevance. For Filtering the top quartile of chains ranked by Localisation score were selected, re-ranked and evaluated by Relevance

Having prepared the networks, we applied the ChainRank method on them. To determine the maximal length parameter for the analysis we took into consideration the distance of the start and end proteins in the GS. For the muscle specific application the canonical distance would be 9, however, due to the differences of the PPI representation of complexes (see in Evaluation of the input network, Fig. 2b) we rationalized using a maximal length 8. For the COPD specific application we used 7 for maximal length, following similar reasoning. In the evaluation process we assessed the improvement of the different scores in finding GS proteins in the top ranked results compared to random prioritisation. We evaluated the performance both by using only individual scores to rank and also by combining the scores. Figure 4 details the dependence of performance on different p-value cut-offs.

For the muscle specific case we ran the ChainRank using the IGF-Akt proximity subnetwork and maximal length 8, retrieved 9351 chains. For the COPD application case (MAPK proximity subnetwork) we computed the chains with maximal length 7, finding 71838 chains. In this case Relevance scores showed high discrepancy from normal distribution therefore the introduced p-value calculation can be misleading for this score. Instead, we show our results by the number of top chains in this scenario.

In the muscle specific scenario, results show that the Connectivity score has the highest improvement of the scores (Fig. 4a). Detailed analysis reveals that this score show especially high improvement with very low p-values however, with growing p-values this improvement quickly decreases to an average of factor 1.8-2 for significant chains. Furthermore, in the top 5 chains Connectivity already finds one of the shortest GS path represented in the input network (Fig. 2c), i.e. IGF1-Akt-mTOR-RPS6KB1. Localisation also introduces an improvement of factor 1.5 amongst the significant chains and maximizes the Recall under 0.001 p-value (Additional file 1: Figure S2). In the MAPK scenario the Relevance score outperformed the other scores showing consistent improvement in top chains (Fig. 4b, Additional file 1: Figure S3). We analyzed the robustness of the algorithm by comparing the performance of Localisation score to TS score in the Muscle specific case and Relevance to Fc score in the COPD specific case. Results showed that the method produces similar improvement for the scores in these scenarios (Additional file 1: Figure S6) and thus it is robust to changes of the scores.

We also investigated the performance of the defined combined strategies. We computed the Combined score as the equal weighted sum of the three normalized scores and evaluated its improvement. With these settings this score could not improve over the best individual scores and therefore we do not report further results. Furthermore, we applied the filtering strategy for both scenarios. For the IGF-Akt case we used a Connectivity filter before evaluating the chains by the Localisation score. We applied a threshold of 0.05 for the filtering. This method introduces a strong and stable increase in improvement (Fig. 4a) which shows good applicability in arbitrary sized subnetwork retrieval. For the MAPK application we investigated the effects of COPD on muscle, therefore we used Localisation filtering and evaluated Relevance on the reduced list of chains. We used the top quartile of the ranked chains to set a filtering threshold. Together with the intersection strategy, in which we applied the same parameters, filtering introduced comparable improvement to Relevance score. To conclude we showed that combining different scores can improve the prediction power of the algorithm and they are capable to mimic complex biological contexts.

**Fig. 5:**
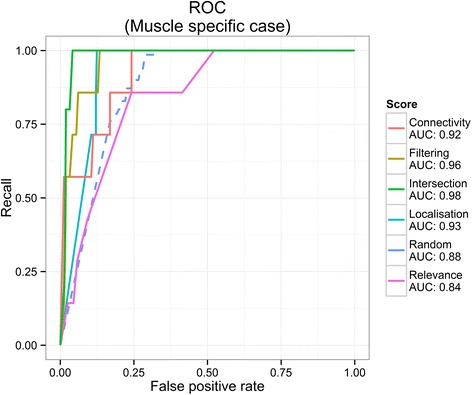
ROC curve and AUC of the muscle specific case. Localisation and its combined scores with Connectivity shows the highest AUC. Random score (*dashed line*) already has an increased performance over completely random guess (*diagonal line*, not shown), which can be accounted for by the constraints introduced by the underlying network topology. AUC values appear below the names of the scores

We evaluated the receiver operating characteristic (ROC) curve and the area under curve (AUC) (Fig. 5) which shows the significant improvement over random scoring. Next, we investigated the effect of the maximal length parameter on the improvement of the chain scores. We found that length does not have a significant effect on the ranking performance (Additional file 1: Figure S4).

**Fig. 6:**
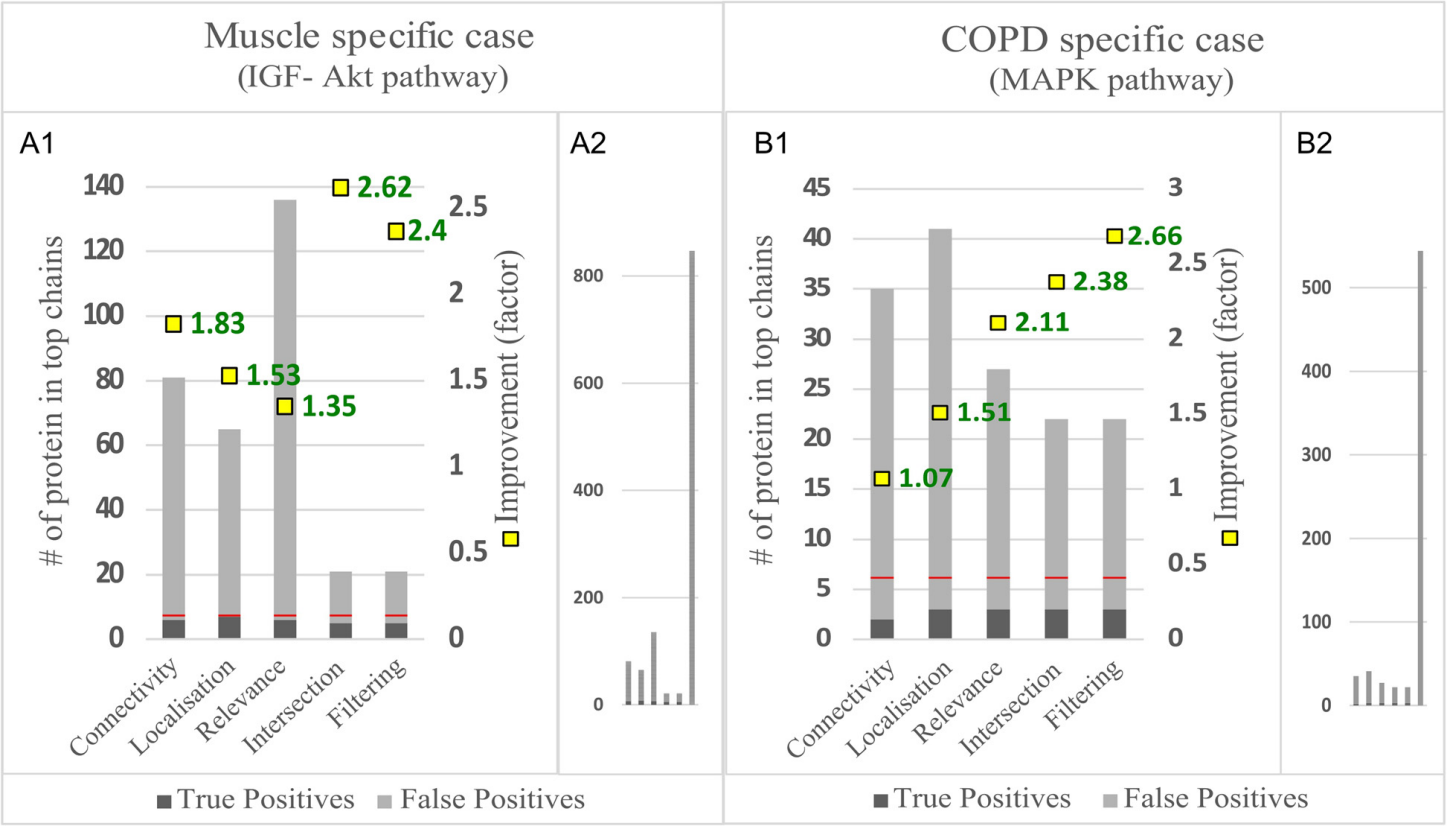
Results of the evaluation after cut-off. (A1) For the muscle specific case 0.015 was used as cut-off threshold. (B1) shows the performance of COPD specific case evaluating the top 50 chains. Red lines show the number of GS proteins (positives), second axis shows the improvement. To represent the enrichment capabilities of the method we compare the recreated pathways to the input network (A2, B2)

**Fig. 7:**
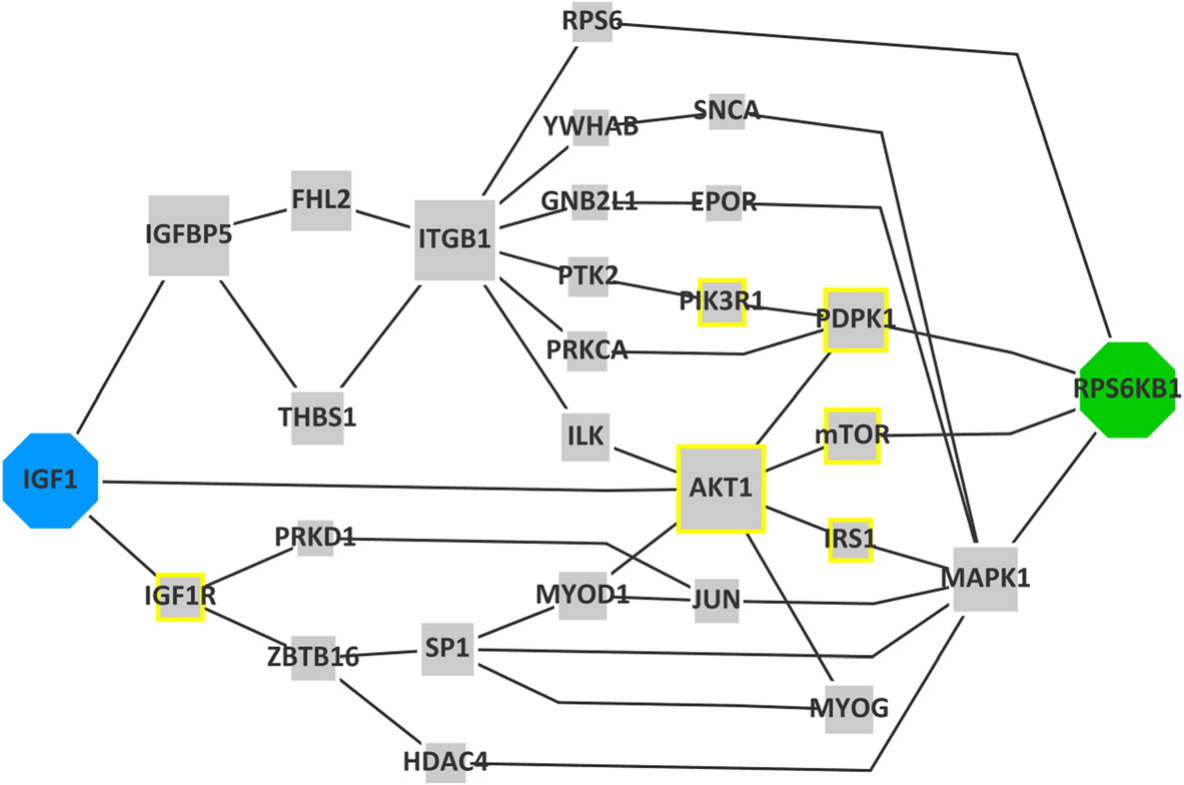
The recreated IGF-Akt pathway. The results of ChainRank were filtered by taking the intersection of the chains that has lower Connectivity and Localisation score p-values lower than 0.05, then the ones with p-values ≤0.015 were assembled into a network (Fig. 6 (a) *Intersection*). The size of the nodes represents the occurrence of a protein in the top chains. Octagons indicate the start and end proteins, nodes with yellow border shows the gold standard proteins

Finally, we identified relevant thresholds that can be used to construct significant subnetworks and recreate the target pathways. Taking into account the improvement-recall trade-off we found a p-value of 0.015 or the number of chains 50 as a good cut-off value. With these thresholds we show high improvement over random in finding targeted GS proteins (Fig. 6). Assembly of chains under the cut-off value shows that the algorithm finds the main chains connecting the targeted start and end proteins and identifies relevant alternative chains with a recall of 67 % and a precision of 30 % (Figs. 6, 7 and Additional file 1: Figures S2, S8). As a further evaluation of the approach, we show that the distribution of scores in the recreated pathways are different from the original network. In the recreated pathways, the distribution means of the simple scores are shifted to higher values. The combined scores can further alter this effect, producing a score distribution that resembles more to the GS (Additional file 1: Figure S5) indicating that the scores indeed capture biological context.

## Discussion and conclusion

In recent decades huge amounts of data have been accumulated in biological research but up to now these valuable data sources remain underutilized in terms of applications for integrative analysis and data mining. Systemic use of biological data could help to create more personalized and contextualized information and overcome the current rigid and generally simplistic representations of mechanisms involved in biological processes and their regulation. This calls for bioinformatics tools that can facilitate data analysis and help in the interpretation of these huge datasets. Biological networks could play an important role in this procedure as they have already shown their utility in many applications. Current high-throughput methods, however, are prone to errors e.g. in yeast two-hybrid systems high false positive rates and platform-specific biases [32] still remain problematic. As a result, inconsistencies could be present in the PPI networks that create alteration in the representation of signalling pathways [4, 33] which our results also confirmed (Evaluation of the input network).

The ChainRank method introduces a data-driven biological search tool that can be applied in widespread research situations. Our goal was to create a tool that can retrieve context specific subnetworks by using different evidences (e.g. expression profile, literature mining). Evaluating a specific application case is a complex task, which we addressed by recreating selected gold standard pathways.

Overall, our evaluation results showed that the generated scores can create domain specific effects. We showed that filtering the chains by scores and intersecting top scoring chains can create improvements in precision and can be applied to simulate complex biological contexts. Although this evaluation is limited only to a few contexts (muscle and COPD) we believe that it gives a representative result to show the general applicability of the method and encourage its usage. Using the three developed ranks, we showed a 50 % improvement (factor 1.5), on average, in the precision of finding gold standard proteins in our top ranked chains. We also showed that combining ranks, for example by pre-filtering with one score before ranking by another, can improve the precision by up to a factor of 2.5. We achieved as high as 11 % improvement in the area under the receiver operating curve (AUC) (Fig. 5) which compares favourably with Bader’s results [34] who reports a similar improvement but with a less generic framework and using protein complexes as a gold standard. Our results are comparable to [12] and [11] who use signal transduction pathways in yeast and human respectively as gold standards and report recall of 50–85 %, and precision of 18–42 %. Therefore, our method generalises the achievements introduced by Scott et al. [11] and Teku et al. [12] by introducing additional, non-expression based evidences and allowing to tune for multiple contexts such as tissue specificity or disease association. We were able to replicate our results with different pathways (IGF-Akt, COPD specific MAPK sub-pathways) and different initial conditions (different input networks). Overall the evaluation showed strong evidence that the method provides improved specificity to generate context-specific networks and therefore supports the viability of the concept.

Although we only showed the applicability of our methodology using PPI networks and in two different contexts (muscle and COPD), it is a generic tool that is applicable for various network types, like metabolic networks or disease networks. Integrated networks incorporating several interactome layers, like proteomics, metabolomics, diseases, etc. can also be used with the method. In addition, scoring criteria can be easily created using various private and public data sources. Although, the new criteria would have to be validated, the accumulation of different context profiles could pave the way for an integrated analysis framework. The differences in performance of individual scores in different biological context (Fig. 4) underscore the importance of appropriate selection of scores depending on the scientific question.

The method can be utilized to analyse many research questions, for example: a) given a set of data-driven associations, e.g. oxidative stress and proteolysis, what is the most likely causal, mechanistic connection in a given context? b) what are the common mechanisms driving different diseases, e.g. systemic effects of COPD and diabetes mellitus type 2? c) can computational modelling be supported by reducing the number of interactions to the biologically most relevant ones and thereby generate manageable complexity [35]? Another promising application field could be the analysis of synthetic lethality in the context of anticancer therapy. By providing evidence-supported alternatives to classical consensus pathways ChainRank could open up new avenues of investigation. A possible avenue is the improvement of the search algorithm for example to use “information propagation” methods [36] to include information from the neighbourhood of a chain into the ranking and thereby see whether biological modularity can be used to further enhance the context specificity of the results. Another interesting aspect would be to implement and compare the current exhaustive search with a heuristic search algorithm that is possibly usable on the full multi-million node and association network that makes up our current biological knowledge.

### Availability

Project home page: https://github.com/atenyi/ChainRank

Programming language: R

## Additional file

Additional file 1:
**This file contains supplementary tables, figures and further information on the scores and the search algorithm.** (PDF 1.43 MB)

## Abbreviations

AUC: area under curve
COPD: Chronic Obstructive Pulmunary Disease
FP: false positive
GS: gold standard
P: positive
PPI: protein-protein interaction
ROC: receiver operating characteristic
TP: true positive
