# Supplementary material for: ChainRank, a chain prioritisation method for contextualisation of biological networks
Source: BMC Bioinformatics. 2016 Jan 5;17:17. doi: 10.1186/s12859-015-0864-x (PMC4700624; doi:10.1186/s12859-015-0864-x)

ChainRank, a chain prioritization method for contextualisation of biological networks

Additional file 1

Ákos Tényi, Pedro de Atauri, David Gomez-Cabrero, Isaac Cano, Kim Clarke, Francesco Falciani, Marta Cascante, Josep Roca, Dieter Maier

Text S1. Search algorithm

The chain search algorithm was implemented to find every non-cyclical path in a network that connects a start and an end set of elements until an added maximal depth. The basic problem is defined by the National Institute of Standards and Technology (NIST) as the “all simple paths” problem which is an NP hard problem because of the exponential number of simple paths. This means that even in a smaller network with few hundreds of nodes the results can exceed millions of paths. We implemented a recursive depth first search (DFS) algorithm that searches all the paths from a start set of nodes until it finds all the paths to an end set of nodes. Furthermore the paths going through start and end vertices are excluded. The output of the algorithm is a list of chains.

Input:

N: Network;

S: Start nodes;

E: End nodes;

MD: Maximal depth,

Output: ChainList = []

```
procedure main(N, S, E,MD):
    ChainList = []
    depth = 0
    for all vertex s in S
        ChainList.append(DFS(N, s, E,MD,depth))
end procedure

procedure DFS(N, s, E,MD,depth):
    Chains = []
    depth = depth + 1
    V = N.adjacentNodes(s)
    V = nonVisited(V)
    for all vertex v in V do
        ChainTail = []
        if v in E
            ChainTail.append(v)
        else if depth > MD  then
            backtrack
        else
            ChainTail.append(DFS(N,w,E,MD,depth))
        Chains.append(ChainTail)
    return Chains
end procedure.
```

Text S2. Localitsation score

Microarray Expression data:
During January 2013 all available samples of the following platforms were identified from Gene Expression Omnibus (1):

a. GPL96: Affymetrix Human Genome U133A Array
b. GPL570: Affymetrix Human Genome U133 Plus 2.0 Array
c. GPL6480: Agilent-014850 Whole Human Genome Microarray

Those platforms were selected because at that time they provided the largest number of human mRNA profiles. The normalised profiles of all samples were downloaded using GEOquery (2). Then the samples were processed in four steps:

a) Each sample was annotated by mapping the meta-data associated to each sample to a controlled vocabulary of muscle and cancer.
b) For each sample the number of missing values were computed and if the number of missing values were larger than 5% the sample was excluded.
c) For each gene (using entrezgene reference) a value was computed as the average of all probes associated to the gene. In each platform the number of genes (ngenes) profiled was constant.
d) For a given platform, each sample was normalised by ranking: the gene with a maximum value was given a value of ngenes and the one with minimum value was set to 1.
e) At the end of the processing and to ensure robustness of the statistics to be computed only those experiments (GSE codes) that contained more than 9 non-filtered samples were included.

Calculation of score
To calculate the score we estimated the mean variability of a gene in muscle and the rest of the body for the different platforms. To this end we considered those samples associated to muscle, not associated to muscle and in all cases we excluded those associated to cancer. Then we estimated for those experiments (GSE codes) with at least 10 samples the ratio of "variability in muscle"/ "variability in non-muscle". Top rank-based genes are expected to be those gene highly varying in the muscle but not in other tissues, therefore we consider those as candidates to be associated to muscle regulation and/or muscle function.

Text S3. Relevance score

To elucidate the transcriptional changes between skeletal muscle of young and elderly populations with a high degree of confidence we performed a meta-analysis of existing microarray data. Datasets representing baseline transcription were downloaded from the Gene Expression Omnibus (Edgar, 2002). Inclusion criteria were: vastus lateralis muscle, pre-intervention, disease-free subjects aged 18-30 or >60 years, and Affymetrix microarray technology. In total 133 high-quality arrays were included (55 young and 78 elderly adults) from 8 different datasets (accession numbers: GSE1428, GSE14901, GSE1786, GSE19420, GSE21496, GSE9419, GSE9676, GSE27536). Data were normalized to eliminate systematic biases introduced by combining expression data from different independent laboratories using ComBat (Johnson, Li, & Rabinovic, 2007). Genes differentially expressed in elderly muscle versus young muscle were identified using Significance Analysis of Microarrays (Tusher, Tibshirani & Chu, 2001, PNAS) with a 1% FDR threshold. To identify important interactions between components of the ageing signature we inferred global gene co-expression networks representing young and elderly muscle using the ARACNE software (Basso et al., 2005). Statistical significant interactions have been selected using a threshold of p<10-9. The two age-specific networks were merged and visualised using the software application Cytoscape (Shannon et al., 2003) . Modules of highly connected genes were identified using the network community detection algorithm Glay (Su, Kuchinsky, Morris, States, & Meng, 2010). Number of genes obtained from young network: 2381. Number of genes obtained from elderly network: 1310.

To obtain the COPD training effect gene list we retrieved the genes included in the correlation network computed in (Turan et. al., 2011). Genes differentially expressed between sedentary and trained subjects in the populations (healthy, COPD) were identified by t-test followed by Benjamimi-Hochberg multiple correction [20] using a false discovery rate (FDR) threshold of q<10%. Using the ARACNE software (Basso et al., 2005) global gene co-expression network was computed and statistical significant interactions have been selected using a threshold of p<10-9. Number of genes obtained: 4918.

To obtain the mouse inactivity muscle wasting gene list significant genes were retrieved from the study Bialek et al., 2011. In the study transcripts were considered to be regulated if the P value based on two-way ANOVA analyses using time and treatment parameters and evaluating treatment was <0.01 and the fold change between any two groups (control, inactive mouse) was >1.5. Using the ARACNE software (Basso et al., 2005) global gene co-expression network was computed and statistical significant interactions have been selected using a threshold of p<10-7. Number of genes obtained: 10557.

Text S4. Tissue Specific score

This score was motivated by the Tissue Specific score (TS score) described on the Human Protein Atlas website. TS score “corresponds to the score calculated as the fold change to the second highest tissue”. The score is calculated using the FPKM values (number of Fragments Per Kilobase gene model and Million reads) (http://www.proteinatlas.org/about/assays+annotation#rna), computed from RNA-seq data gained from 32 tissues (available at http://www.proteinatlas.org/about/download). The threshold level to detect presence of a transcript for a particular gene is defined as > 1 FPKM on the Protein Atlas website, and thus we set the minimal score to 1 to avoid infinite scores (i.e. protein is not detected in other tissues). We handled missing values by substituting them by the mean of the score in the network.

Table S1. Elements of the EGF-PI3K and ROS-TGFa-EGFR COPD specific MAPK pathways used for the evaluation. Genes with red font color are not part of the canonical MAPK pathway,

Protein name	Related Gene
**ADAM metallopeptidase domain 17 preproprotein**	ADAM17
**v-akt murine thymoma viral oncogene homolog 1**	AKT1
**epidermal growth factor (beta-urogastrone)**	EGF
**epidermal growth factor receptor isoform a**	EGFR
**erbB-2 isoform b**	ERBB2
**coagulation factor II precursor**	F2
**FK506 binding protein 12-rapamycin associated protein 1**	FRAP1
**v-Ha-ras Harvey rat sarcoma viral oncogene homolog isofrom 1**	HRAS
**mitogen-activated protein kinase kinase 1**	MAP2K1
**mitogen-activated protein kinase kinase 2**	MAP2K2
**mitogen-activated protein kinase 1**	MAPK1
**mucin 2**	MUC2
**mucin 5AC**	MUC5AC
**phosphoinositide-3-kinase, catalytic, gamma polypeptide**	PIK3CG
**v-raf-1 murine leukemia viral oncogene homolog 1**	RAF1
**Ras homolog enriched in brain**	RHEB
**ribosomal protein S6 kinase, 70kDa, polypeptide 1**	RPS6KB1
**Sp1 transcription factor**	SP1
**transforming growth factor, alpha**	TGFA
**tuberous sclerosis 1 protein isoform 2**	TSC1
**tuberous sclerosis 2 isoform 4.**	TSC2

Table S2. Detailed overview of the analysed networks and gold standards. The two application cases are detailed in the table. Different network selection strategies were tried out (Section 3.2). Less connected proteins were preferred in the hub reducing case and more connected proteins were preferred in the hub enriching case. The gold standards are also shown in the table. The last column compares the representation of the GS nodes and edges in the Human PPI network and in the reduced networks. The table shows that there is a noticeable difference in the size of the selected networks, when using hub enriching and hub reducing strategies. This noticeable difference is due to the fact that the hub enriched networks showed higher complexity during the chain search, i.e. we found much more chains in the similar sized hub enriching networks than the hub reducing ones.

Application case	Type of network reduction	Network properties		Gold standard (GS)	Start protein	End protein	GS representation (after/before network selection)	
		Number of edges	Number of nodes				Nodes	Edges
**IGF-Akt proximity subnetworks**	Hub enriching	865	314	IGF-Akt pathway	IGF1	RPS6KB1	9/13	10/20
	Hub reducing	2874	1215				11/13	11/20
**MAPK proximity subnetworks**	Hub enriching	583	156	CODP specific MAPK	EGFR	SRF, CREBBP, ELK1, MYC	7/21	4/34
	Hub reducing	1806	1076				12/21	0/34

Table S3. Performance of ChainRank. We assessed the performance of our algorithm by running it with different maximal length parametrization and with different network selection strategies (Section 3.2). Less connected proteins were preferred in the hub reducing case and more connected proteins were preferred in the hub enriching case. The table shows the number of chains found and the time needed for the search for the different networks, and different maximal lengths. The time shown in the table only includes the time consumption of the search in the indicated networks. It does not include the search in the random networks for the p-value calculation. For the test a computer with 2.4GHz processor was used. In the COPD related case the search from the start node to separate end nodes was run parallelly.

Application case	Type of network reduction	Network properties		Max length	Chains	Time
		Number of edges	Number of nodes			
**IGF-Akt proximity subnetworks**	Hub enriching	865	314	6	146	15s
				7	823	1min 15s
				8	9351	15min
				9	70703	1.5h
	Hub reducing	2874	1215	6	162	16s
				7	1146	5min
				8	9078	16min
**MAPK proximity subnetworks**	Hub enriching	544	152	6	11967	2min
				7	71838	47min
	Hub reducing	1806	1076	6	289	4s
				7	765	10s

Table S4. Comparison of improvement in different networks.

Application case	Network reduction type	Threshold (p-value or n)	Connectivity	Relevance	Locality	Intersection	Filtering
**IGF-Akt proximity subnetworks**	hub enriching	0.015	1.83	1.35	1.54	2.83	2.47
	hub reducing	0.015	0.45	1.06	1.43	1.67	1.67
**MAPK proximity subnetworks**	hub enriching	50	1.07	2.11	1.50	2.38	2.66

Figure S1. Distribution of scores in the input subnetwork. Score distributions were plotted in the whole PPI network, the pathway specific networks and the recreated pathways. The Connectivity score distributions shows the effect of the subnetwork selection step, enriching highly connected proteins. The other two scores' distribution remained intact from the subnetwork selection step.

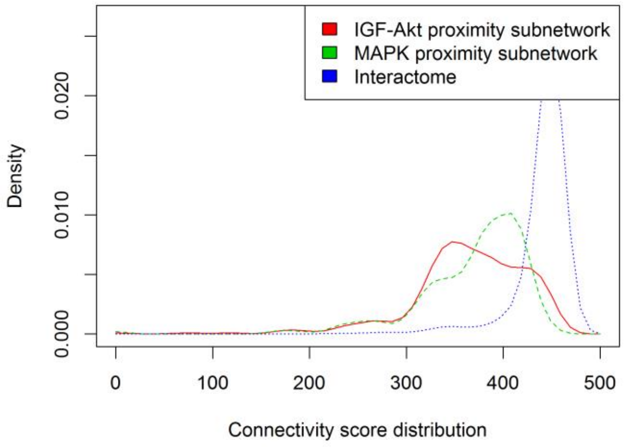

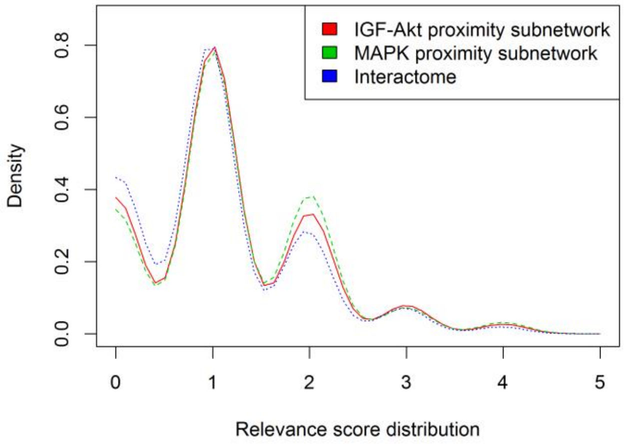

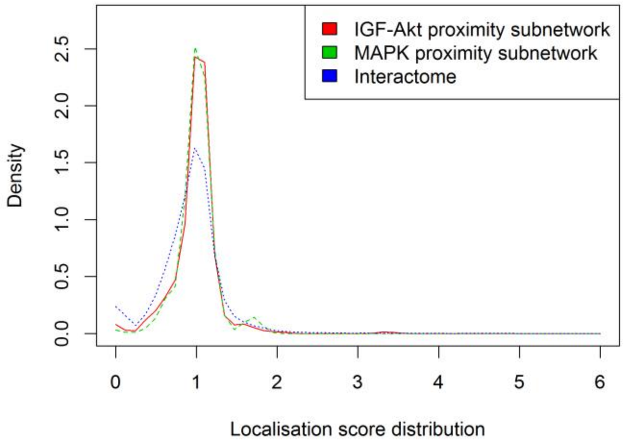

Figure S2. IGF-Akt, muscle specific case, maximal length 8, detailed (A) Precision and (B) true positive rate. Dashed line shows the random score. On figure (A) number of chains indicated at p= 0.015 and p= 0.05.

A

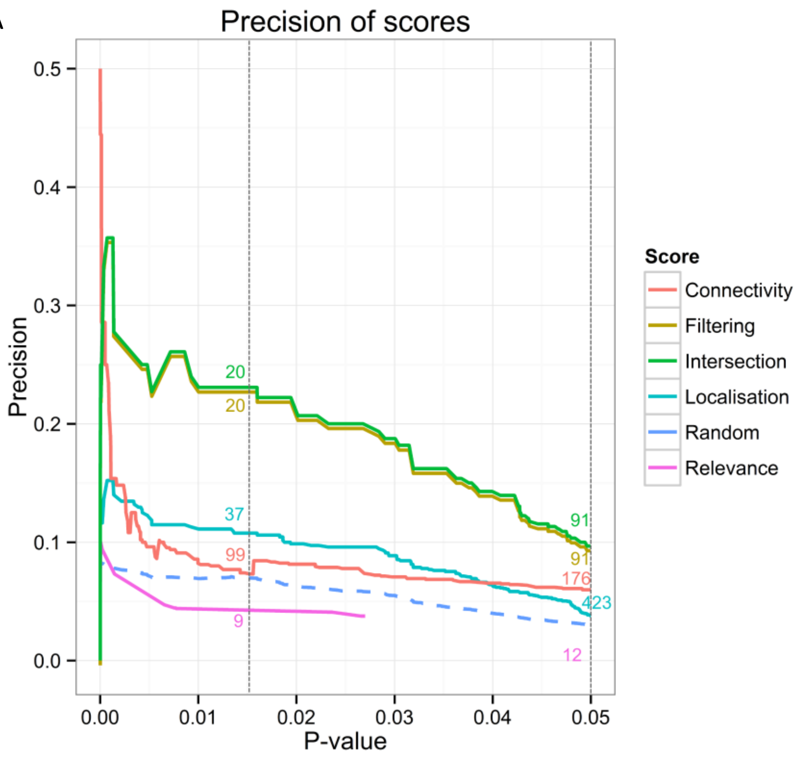

B

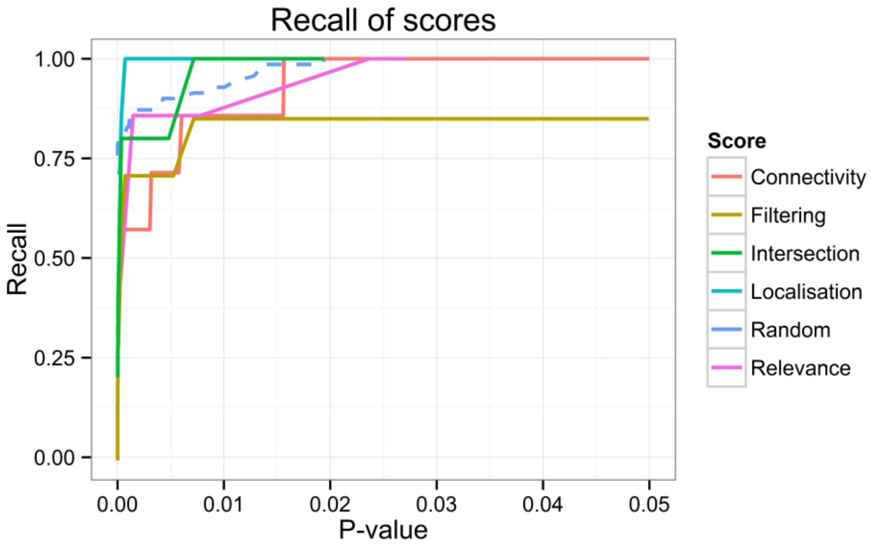

Figure S3. MAPK, disease specific case, maximal length 7, detailed (A) precision and (B) true positive rate (C) ROC curve and AUC. Dashed line shows the random score.

A

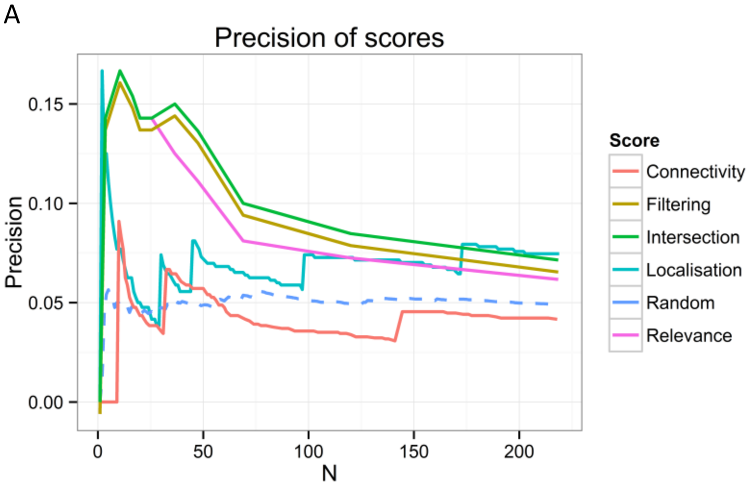

B

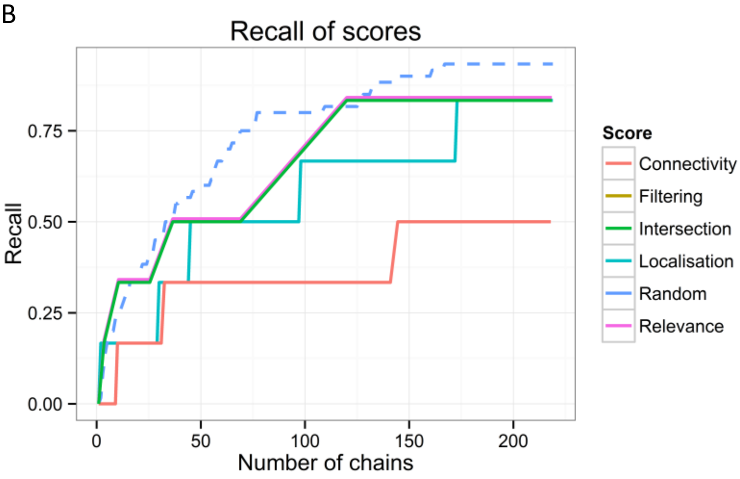

C

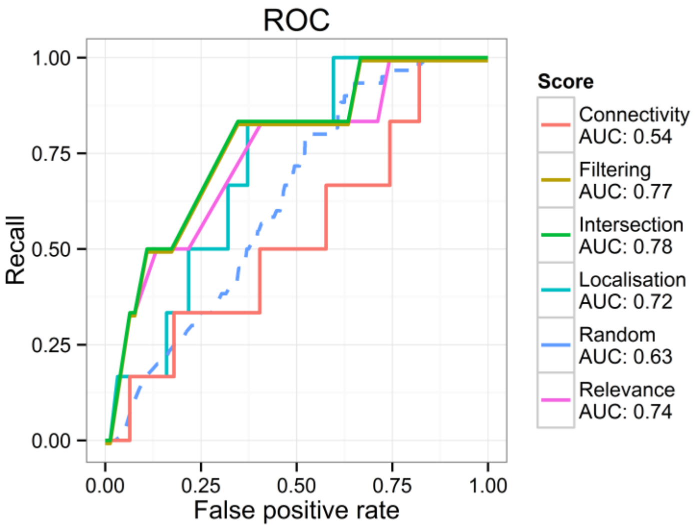

Figure S4. Evaluation of the effect of maximal length on improvement. (A) shows the muscle specific case (IGF-Akt gold standard) with the comparison of two networks computed with ChainRank using the parameter of maximal length 7, 8 and 9. (B) similarly, it shows for MAPK application case, with maximal length 6 and 7.

A

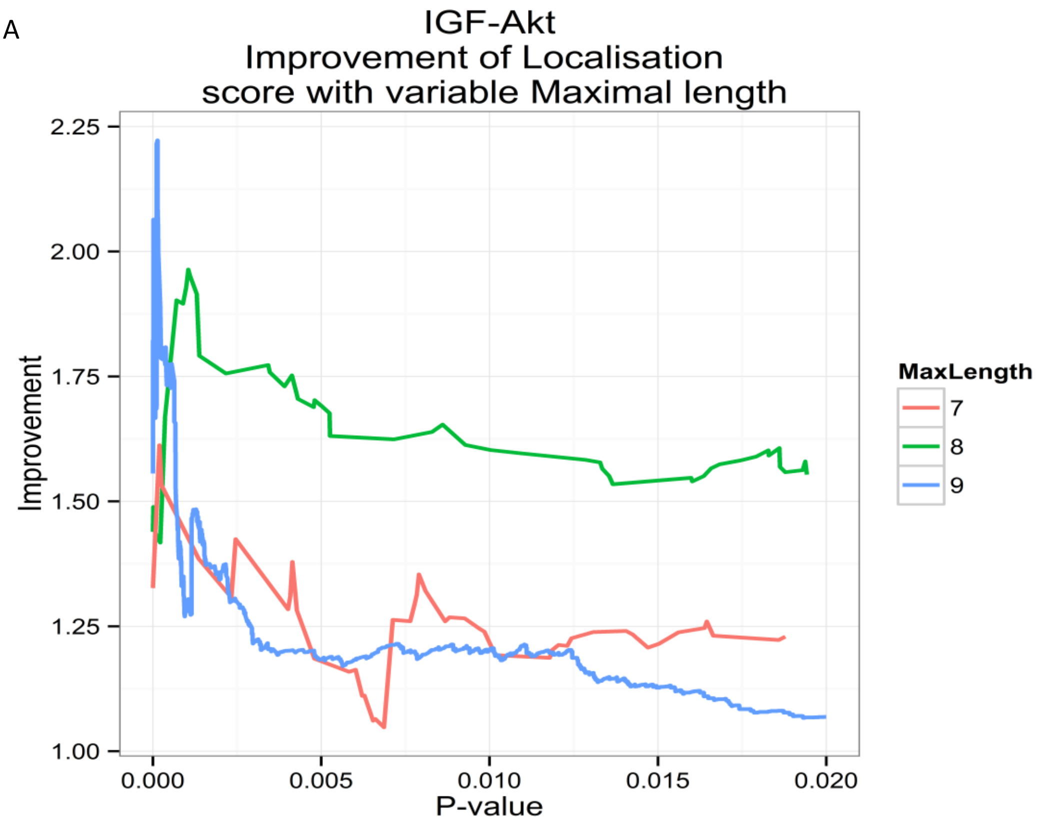

B

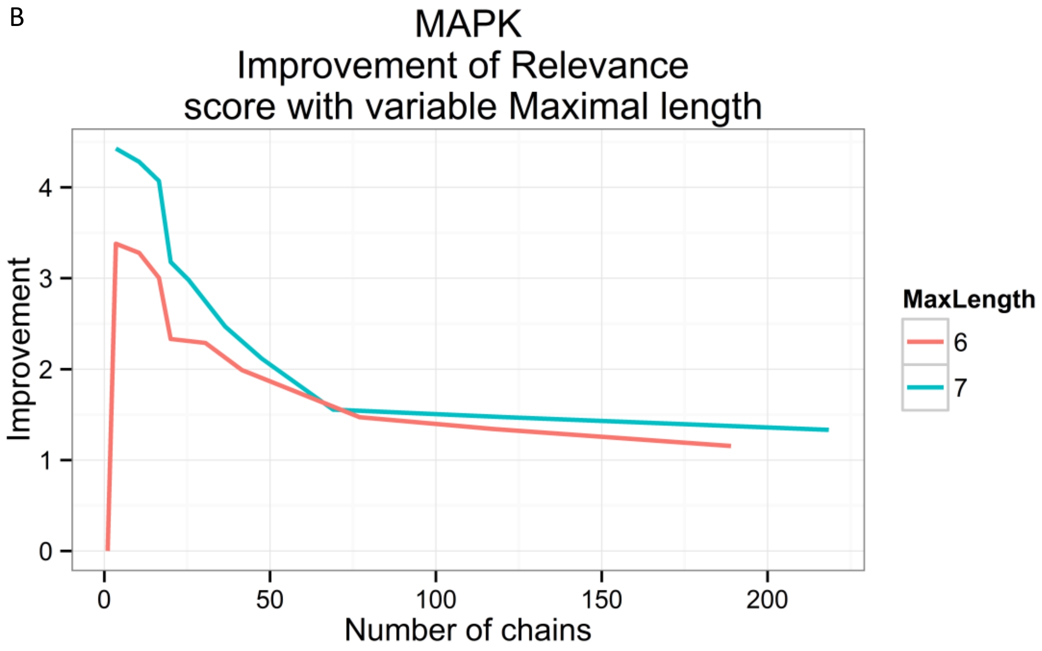

Figure S5. Distribution of scores in the recreated pathways. Score distributions were plotted in the whole PPI network (Interactome), the gold standard pathways and the recreated pathways with the case specific score and the Intersection score specified as Fig. 4 in the main text.

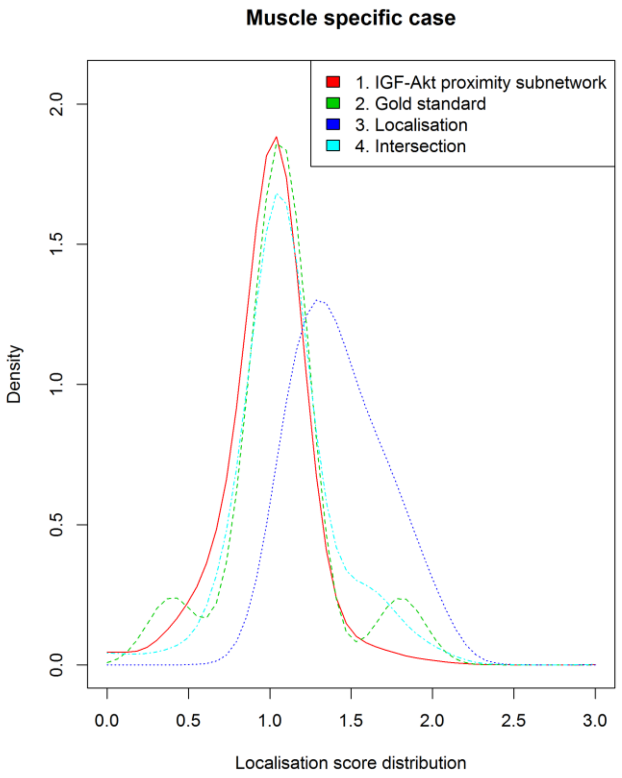

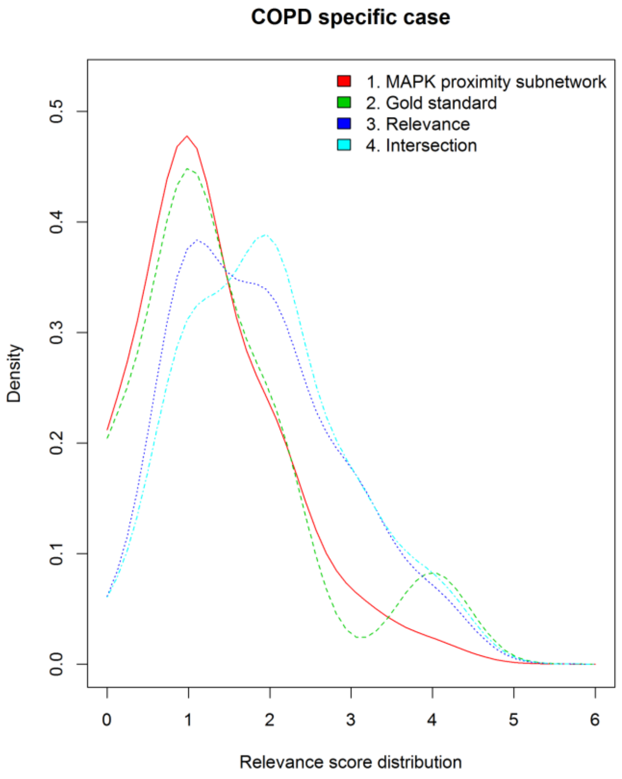

Figure S6. Evaluation of the improvement of the additional scores in the muscle (A) and disease specific (B) cases showing the robustness of the method to different scores. We note that results might be slightly different from Fig. 4 in the main text due to random processes used in the validation.

A

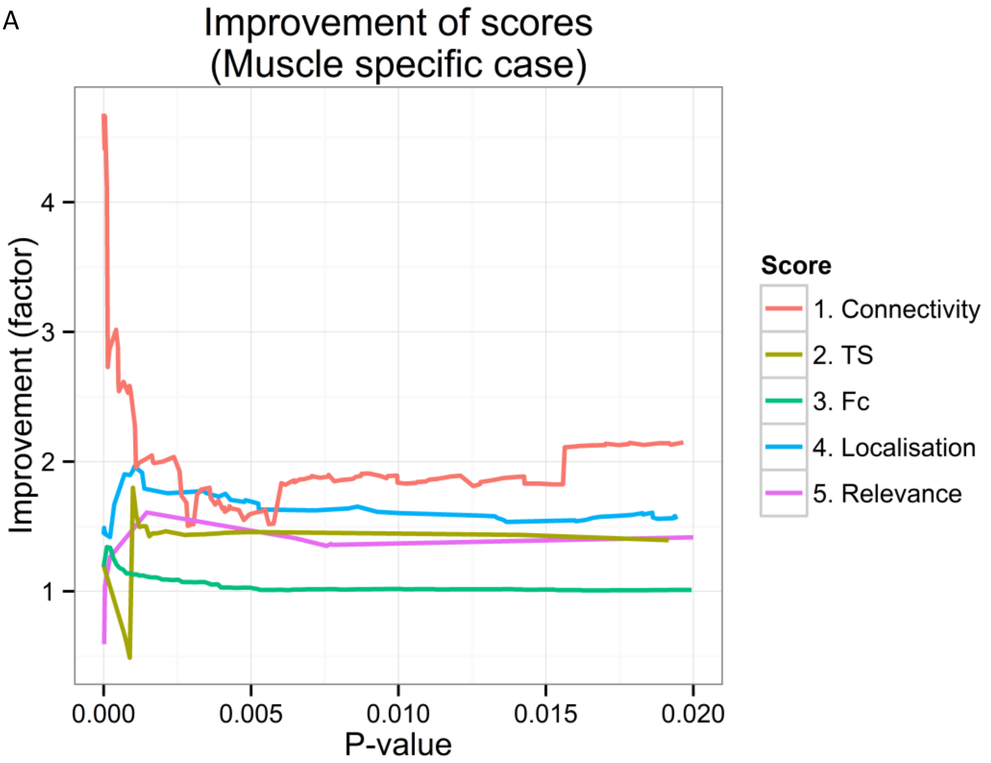

B

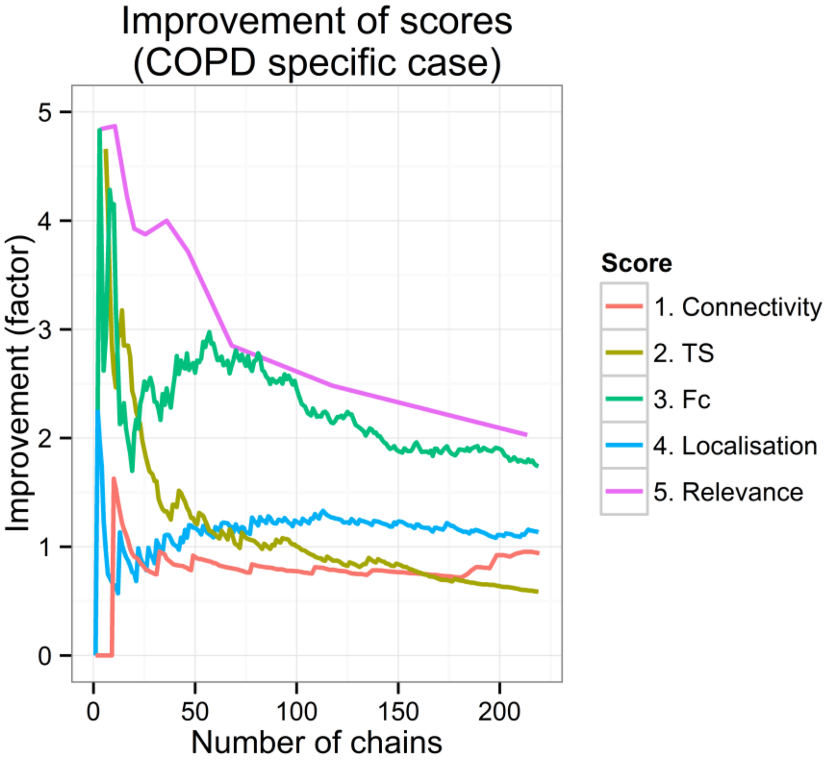

Figure S7. The EGF-PI3K and ROS-TGFa-EGFR COPD specific MAPK pathways used for the evaluation

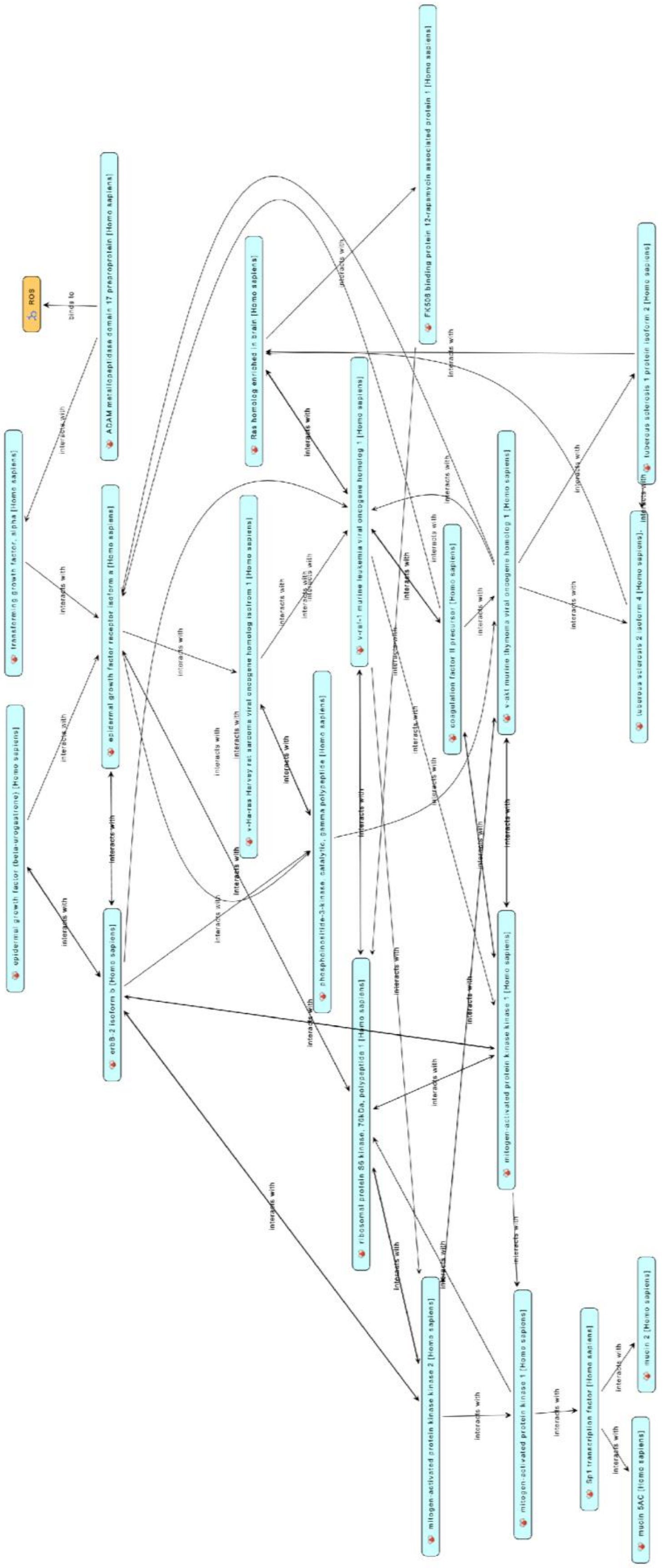

Figure S8. Network of top chains for the IGF-Akt scenario. Chains having a Localisation p-value lower than 0.015 assembled to a network. The networks were created using the network output file of ChainRank by visualizing it in Cytoscape and ordering it by hierarchical layout.

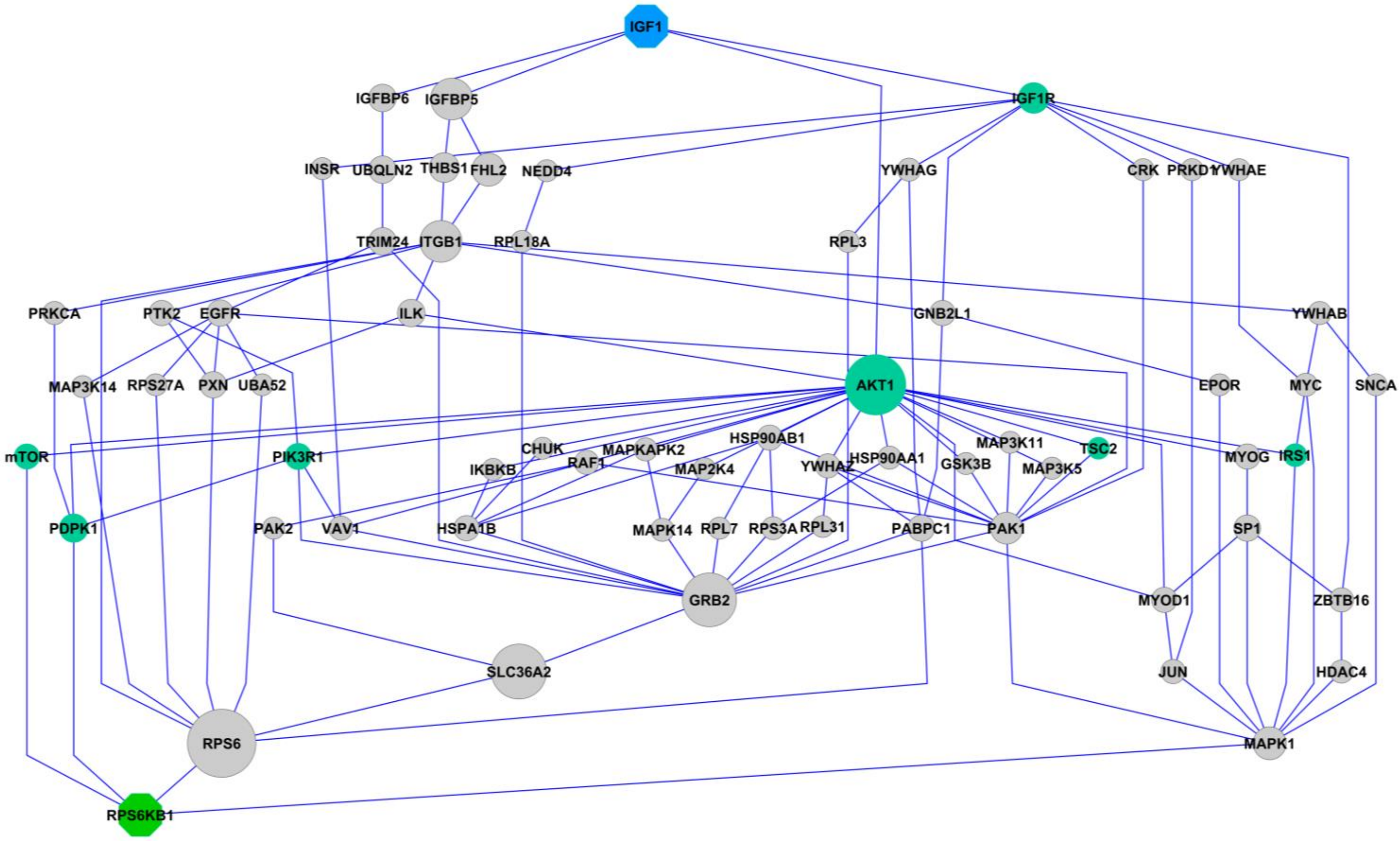

Supplement: Additional file 1: — This file contains supplementary tables, figures and further information on the scores and the search algorithm. (PDF 1.43 MB) [file 12859_2015_864_MOESM1_ESM.pdf]
